# Academic Gratitude as a Mediator between Psychological Well-Being and Academic Buoyancy among college students in West Java

**DOI:** 10.3389/fpsyg.2025.1663694

**Published:** 2026-01-21

**Authors:** Nurul Wardhani, Hendriati Agustiani, Ida Juliana Hutasuhut, Ahmad Gimmy Prathama Siswadi

**Affiliations:** 1 Faculty of Psychology, Universitas Padjadjaran, Jatinangor, Sumedang, Jawa Barat, Indonesia; 2 Faculty of Cognitive Sciences and Human Development, Universiti Malaysia Sarawak, Kota Samarahan, Sarawak, Malaysia

**Keywords:** Psychological Well-Being, Academic Gratitude, Academic Buoyancy, mediation analysis, university students, Indonesia

## Abstract

**Introduction:**

This study aims to examine the relationships among *Psychological Well-Being*, *Academic Gratitude*, and *Academic Buoyancy* among students in Indonesia, and to assess whether Academic Gratitude acts as a partial mediator in these relationships.

**Methods:**

A total of 407 students (304 females and 103 males) participated in an online survey, selected via *stratified random sampling by* academic year. The data were analyzed using a covariance-based structural equation model (SEM).

**Results:**

The results showed that *Psychological Well-Being* was positively related to *Academic Gratitude* and *Academic Buoyancy*, while *Academic Gratitude* was also positively related to *Academic Buoyancy*. Mediation analysis using bootstrapping with 5,000 resamples showed that *Academic Gratitude partially mediates* the relationship between *Psychological Well-Being* and *Academic Buoyancy*, suggesting that gratitude functions as an affective-cognitive pathway that explains the interrelationships among the three constructs.

**Discussion:**

Given the cross-sectional nature of the study design, the direction of the relationship cannot be concluded as causal and requires further examination in longitudinal or experimental studies. Overall, these findings provide empirical support for the role of gratitude in understanding student adaptation to academic challenges in the context of higher education in Indonesia.

## 1 Introduction

Students at various universities in Indonesia are reportedly facing high academic pressure that affects their Psychological Well-Being, including students from the university where this research was conducted. A number of studies show that stress, anxiety, and decreased mental well-being are common problems among students, especially since the COVID-19 pandemic (Karimah et al., 2024; Romadhona et al., 2021). This increase in academic pressure highlights the importance of understanding the psychological factors that help students adapt effectively. Students’ ability to recover from daily academic difficulties—known as Academic Buoyancy—is an important skill for maintaining engagement and learning performance (Martin and Marsh, 2008; Putwain and Wood, 2023).

One psychological factor that can support academic resilience is *Psychological Well-Being* (PWB), which includes self-acceptance, personal growth, positive interpersonal relationships, environmental mastery, autonomy, and life purpose (Ryff, 1995). Students with higher levels of PWB tend to have more adaptive emotional regulation, more stable self-perception, and more focused academic motivation (Allan and McKenna, 2022; Chye et al., 2024). These characteristics can help them respond to academic challenges more constructively. PWB reflects adaptive functioning, including emotional regulation, stability of self-perception, and the capacity to constructively interpret academic experiences. Individuals with high PWB tend to demonstrate more effective problem-solving and stress regulation skills (Ryff, 1995).

In addition to PWB, *gratitude* is an important construct related to positive emotions, academic adaptation, and perceptions of support in higher education. Gratitude is known to increase cognitive flexibility, reduce stress, strengthen interpersonal relationships, and foster a positive orientation toward academic experiences (Cui et al., 2023; Zhen et al., 2021). *Gratitude* is a person’s general tendency to recognize and respond with feelings of thankfulness to the kindness of others who play a role in the positive experiences and outcomes they obtain (McCullough et al., 2002). In higher education, Academic Gratitude can be expressed through appreciation for support, learning opportunities, and various beneficial academic experiences (Bilong et al., 2021; Zainoodin et al., 2021).

The relationship between PWB, *Academic Gratitude*, and *Academic Buoyancy* can be understood through the *Broaden-and-Build theoretical* framework (Fredrickson, 2001), which explains that positive emotions are associated with the expansion of cognitive capacity and attention, as well as the development of long-term psychological resources. In this perspective, PWB can be associated with the emergence of positive emotions, such *as gratitude*, while *gratitude* itself is related to various indicators of academic adaptation. Therefore, gratitude is considered an affective-cognitive pathway that can conceptually help explain the relationship between PWB and *Academic Buoyancy*.

Although *gratitude* has been extensively studied in relation to well-being and various aspects of academic functioning, research directly examining its role as a mediator in the relationship between PWB and *Academic Buoyancy remains* limited, including in Indonesia. This research gap is important given the need to understand the psychological mechanisms that help students cope with the challenges of academic pressure. In addition, research on gratitude in the Indonesian context remains relatively limited, so further empirical evidence is needed to understand how this positive emotion operates in higher education settings.

*Academic Buoyancy* and *Academic Resilience* are interrelated but differ in the scope of academic adaptation. *Academic Resilience* refers to students’ ability to endure and recover from severe and prolonged academic stress or difficulties, such as major failures or challenging life circumstances. On the other hand, *Academic Buoyancy* describes the ability to overcome mild to moderate daily academic obstacles, such as unsatisfactory test scores or routine assignments (Martin and Marsh, 2008). A number of studies show that *Academic Buoyancy* can be considered a more immediate and operational form of resilience in daily learning, as well as the basis for the development of long-term *Academic Resilience* (Martin and Marsh, 2008; Putwain et al., 2012). Therefore, both have similar adaptive characteristics but differ in the intensity of the challenges faced. Therefore, both share similar adaptive characteristics but differ in the intensity of the challenges they face.

Based on the theoretical framework and previous empirical findings, this study tests four hypotheses:

1. PWB is positively related to *Academic Buoyancy*;
2. PWB is positively related to *Academic Gratitude*;
3. *Academic Gratitude* is positively related to *Academic Buoyancy*; and
4. *Academic Gratitude* mediates the relationship between PWB and *Academic Buoyancy*.

**Figure 1:**
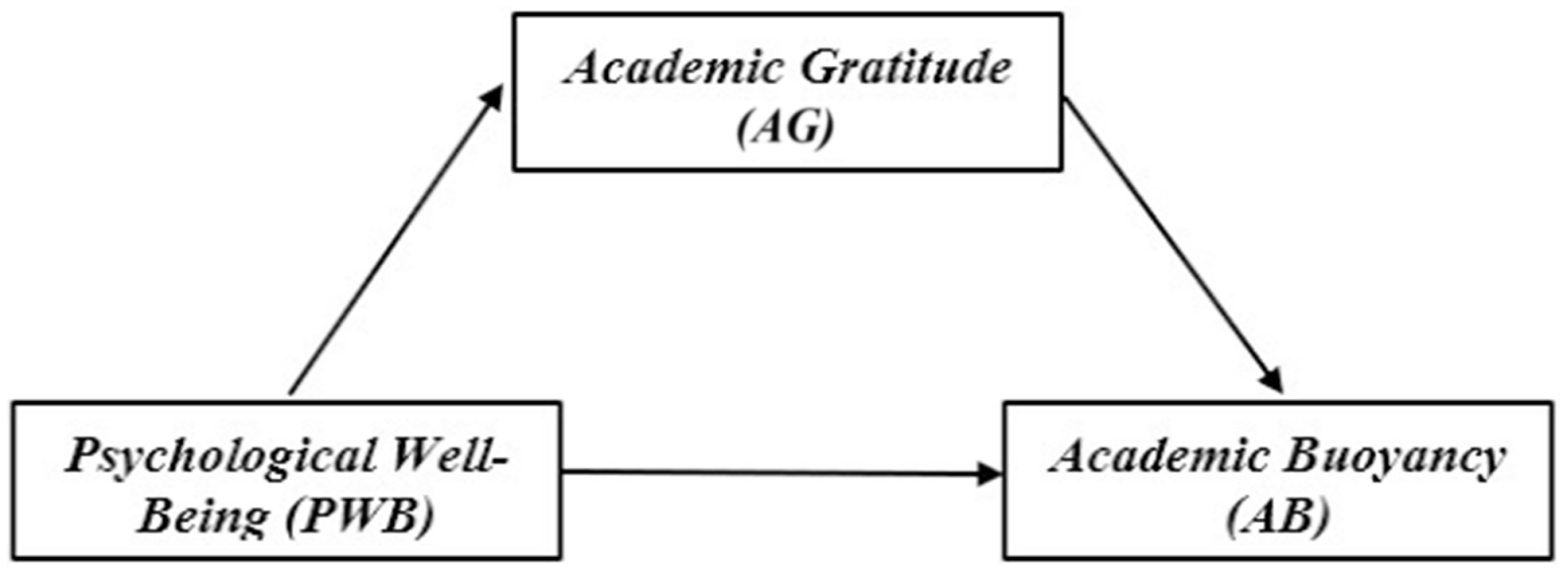
Conceptual model illustrating the hypothesized relationships among Psychological Well-Being (PWB), Academic Gratitude (AG), and Academic Buoyancy (AB).

Figure 1 shows a conceptual model illustrating the relationships between the variables tested.

## 2 Method

### 2.1 Research design

This study uses a *cross-sectional* quantitative survey design to examine the relationships among *Psychological Well-Being*, *Academic Gratitude*, and *Academic Buoyancy*. Figure 1 *presents* a conceptual model of the relationships among the variables tested in this study.

### 2.2 Participants

This study involved 407 undergraduate students, representing 2.2% of the total 22,572 students enrolled at a state university in West Java. Participants were selected through stratified random sampling based on year of enrollment to ensure a representative sample. Multivariate outlier checks using Mahalanobis Distance (MD) identified three data points as outliers, which were excluded from the analysis.

The final sample consisted of 304 females and 103 males, with distribution based on year of study: first year (88), second year (118), third year (96), and fourth year (105).

Inclusion criteria included active undergraduate students aged 18–25 years enrolled in the current semester who provided consent to participate. Exclusion criteria included questionnaire completion rates below 80%, invalid response patterns (straight-lining), and inactive student status.

The sample size was determined by specific guidelines *for covariance-based structural equation modeling* (SEM), namely the ratio of estimated parameters to the number of respondents. According to Bentler and Chou (1987), the recommended minimum sample size is 5 times the number of parameters and can be increased to 5–10 times that number, as recommended by Gye-Soo (2016). This research model includes 52 parameters, so the minimum sample size required is 260 respondents. With 407 respondents, the research sample size meets and exceeds the general SEM recommendation of >300 respondents for stable and reliable model estimation (Kline, 2023).

### 2.3 Instruments

This study uses three main instruments to measure *Psychological Well-Being*, *Academic Gratitude*, and *Academic Buoyancy*. All instruments have undergone linguistic and cultural adaptation in accordance with psychological scale adaptation guidelines.

#### 2.3.1 Psychological Well-Being

*Psychological Well-Being* was measured using the Indonesian adaptation of *the Psychological Well-Being Scale*, which covers six main dimensions: autonomy, positive relationships, environmental mastery, self-acceptance, personal growth, and life purpose (Ryff, 1995). The adaptation was carried out through a forward-backward translation procedure, followed by assessment by three psychology experts from Universitas Padjadjaran, Jatinangor, Indonesia (Unpad), Universitas Indonesia, Depok, Indonesia (UI), and Universiti Malaysia Sarawak, Kota Samarahan, Sarawak, Malaysia (UNIMAS). A trial involving 30 students was conducted to ensure clarity of language and understanding of the scale items. Reliability was measured using Cronbach’s Alpha and Composite Reliability (CR), while convergent and discriminant validity of the was evaluated through Average Variance Extracted (AVE) and Heterotrait–Monotrait Ratio (HTMT) (Hair et al., 2019).

#### 2.3.2 Academic Gratitude

*Academic Gratitude* was measured using *the Academic Gratitude Scale*, a contextual adaptation of *the Gratitude Questionnaire-6* (McCullough et al., 2002). The items were adapted to reflect students’ tendency to acknowledge and respond with gratitude to the meaningful experiences and contributions of others in their academic journey. The adaptation process involved *forward-backward translation and* panel discussions with psychology experts from Unpad, UI, and UNIMAS, as well as preliminary testing with 30 students to ensure linguistic and conceptual equivalence. This scale uses a seven-point Likert format. Validity and reliability were measured using Cronbach’s Alpha, CR, AVE, and HTMT.

#### 2.3.3 Academic Buoyancy

*Academic Buoyancy* was measured using the *Academic Buoyancy* Scale (Martin and Marsh, 2008), which assesses students’ ability to cope with daily academic challenges. This measurement tool was adapted into Indonesian through *forward-backward translation* and evaluated by three psychology experts from Unpad, UI, and UNIMAS to ensure conceptual and linguistic equivalence. A pilot test was conducted with 30 students to evaluate item understanding. Reliability and construct validity were evaluated using Cronbach’s Alpha, CR, AVE, and HTMT.

### 2.4 Data collection procedures

Data was collected online via Google Forms after obtaining ethical approval from the University Ethics Committee. The questionnaire link was distributed to participants via campus email, official academic communication groups, and social media such as Line and WhatsApp. Before participating, participants received an explanation of the research’s purpose, data confidentiality, and the right to withdraw at any time. Participation was voluntary, and no incentives were given. Online surveys were chosen for their efficiency, although the potential for general and social method biases was still taken into account in interpreting the results.

### 2.5 Data analysis

Data analysis was performed using SPSS version 26 and LISREL version 8.80 through *a covariance-based structural equation modeling* (SEM) approach. The analysis stages included reliability checks, construct validity evaluation, measurement model testing, and structural model testing.

Construct validity was evaluated through Confirmatory Factor Analysis (CFA) based *on standardized factor loadings*, Composite Reliability (CR), Average Variance Extracted (AVE), and discriminant validity through the Heterotrait–Monotrait Ratio (HTMT). Internal reliability was evaluated using Cronbach’s Alpha and CR, with values≥ 0.70 as the acceptance criterion. Convergent validity was considered fulfilled if the AVE value was ≥ 0.50.

After the measurement model met these criteria, hypothesis testing was conducted using SEM. Model fit was evaluated using the χ^2^/df index, RMSEA, CFI, TLI, and SRMR, with thresholds referring to Hair et al. (2019). The list of indices and acceptance criteria is presented in Table 2 in the Results section.

**Table 2:** Model fit indices for the structural model, including absolute, incremental, and parsimonious fit measures alongside their recommended cut-off criteria.

Index	Value	Criteria
χ^2^/df	3.26	> 3 and ≤ 5 (acceptable)
RMSEA	0.075	≤ 0.08 (acceptable)
SRMR	0.084	≤ 0.08 (slightly above the ideal cutoff)
TLI	0.95	≥ 0.90 (good)
CFI	0.95	≥ 0.90 (good)

Mediation analysis was performed using the bootstrapping procedure with 5,000 resamples to obtain more stable estimates of *indirect effects*. Mediation effects were considered significant if the 95% confidence interval did not include zero or if the *p*-value was < 0.05 (Hayes, 2022).

## 3 Results

### 3.1 Construct validity and reliability

Evaluation of the measurement model showed that all constructs had adequate validity and reliability. All retained indicators had *standard factor loadings* (*λ*) ≥ 0.50, meeting the minimum threshold for indicator validity.

The Average Variance Extracted (AVE) values ranged from 0.50 to 0.67; thus, convergent validity was established for all constructs. In addition, the Composite Reliability (CR) was also strong (0.85–0.97), indicating good internal consistency (Hair et al., 2019).

Discriminant validity was tested using the Heterotrait–Monotrait Ratio (HTMT). The HTMT values for most construct pairs were below 0.85. However, the HTMT value for the PWB–AG pair was slightly above 1.0, suggesting potential construct overlap. In addition, the use of self-report instruments can increase *method* var*iance*. Both of these issues are discussed further in the limitations section.

A summary of construct validity and reliability is presented in Table 1.

**Table 1:** Construct reliability and validity indices for all variables included in the study.

Construct	Cronbach’s Alpha	CR	AVE	HTMT
*Psychological Well-Being*	0.92	0.97	0.58	1.08
*Academic Gratitude*	0.75	0.85	0.50	1.08
*Academic Buoyancy*	0.59	0.89	0.67	0.72

### 3.2 Confirmatory factor analysis (CFA)

The measurement model shows *acceptable* fit, although some indices (e.g., SRMR) are slightly above the ideal range, necessitating careful model interpretation. The χ^2^/df value of 3.26 and RMSEA of 0.075 are within *the acceptable* range *of fit*, while CFI and TLI are 0.95, respectively, indicating a good level of model fit. The SRMR value of 0.084 is slightly above the ideal limit (< 0.08), but is still within the acceptable tolerance range in SEM analysis (Hair et al., 2019).

The complete model fit indices are presented in Table 2.

### 3.3 Structural equation modeling (SEM)

Structural model analysis shows that all paths between variables are statistically significant and consistent with the research hypotheses.

The results of hypothesis testing are as follows:

- H1: PWB → AB (*β* = 0.50, *t* = 8.37, *p* < 0.001).
- H2: PWB → AG (*β* = 0.44, *t* = 7.86, *p* < 0.001).
- H3: AG → AB (*β* = 0.33, *t* = 5.74, *p* < 0.001).
- H4: The mediating effect of AG is significant based on bootstrapping (*p* < 0.05).

**Figure 2:**
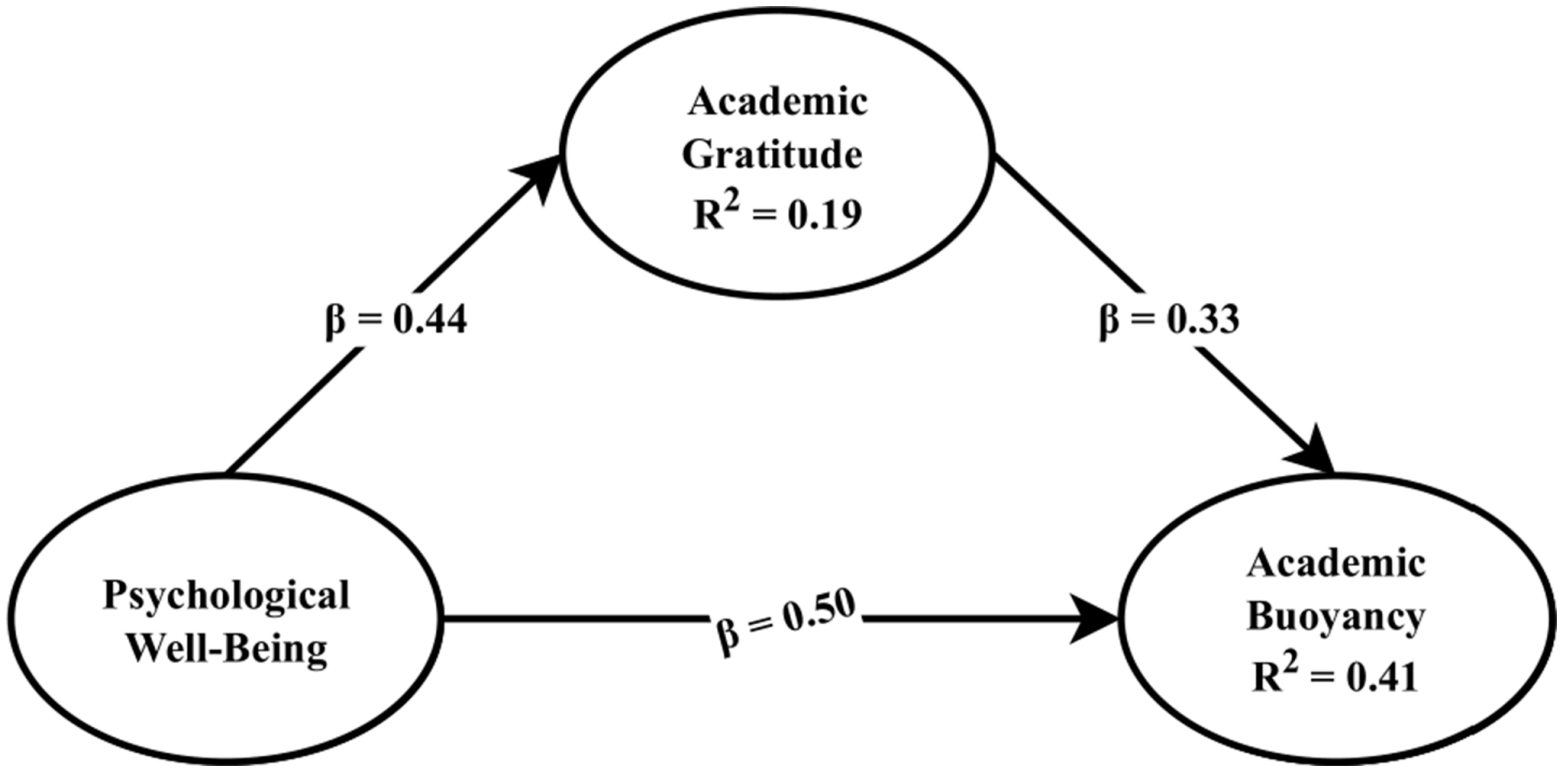
Structural equation model with standardized path coefficients for the relationships among Psychological Well-Being (PWB), Academic Gratitude (AG), and Academic Buoyancy (AB).

A summary of the structural path results is presented in Table 3, and an illustration of the structural model is shown in Figure 2.

**Table 3:** Path coefficients of the structural model, including standardized estimates (β), *t*-values, and significance levels.

Path	*β*	*t*-value	*p*-value
PWB → AG	0.44	7.86	< 0.001
PWB → AB	0.50	8.37	< 0.001
AG → AB	0.33	5.74	< 0.001

Meanwhile, the coefficients of determination (R^2^) for the Academic Gratitude and Academic Buoyancy variables are shown in Table 4.

**Table 4:** Coefficients of determination (*R*^2^) for the endogenous variables, indicating the proportion of variance in Academic Gratitude and Academic Buoyancy explained by the predictors in the structural model.

Variable	*R* ^2^
Academic Gratitude	0.19
Academic Buoyancy	0.41

### 3.4 Mediation analysis

The mediating effect of *Academic Gratitude* was tested using the bootstrapping procedure (5,000 *resampling*). The results of the analysis showed that the indirect path from *Psychological Well-Being (PWB)* to *Academic Buoyancy (AB)* through *Academic Gratitude (AG)* was significant (*p* < 0.05) (Hayes, 2022). The results show that the indirect path PWB → AG → AB is significant (*p* < 0.05), indicating partial mediation. Thus, *Academic* Gratitude functions as an additional affective-cognitive pathway that helps explain the relationship between *Psychological Well-Being* and *Academic Buoyancy*.

## 4 Discussion

This study aims to explore the relationship between *Psychological Well-Being*, *Academic Gratitude*, and *Academic Buoyancy* among students at a university in West Java. The following discussion is based on four research hypotheses and integrated with previous empirical findings and relevant theoretical frameworks.

### 4.1 H1—*Psychological Well-Being* and *Academic Buoyancy*

The results of this study indicate a positive association between *Psychological Well-Being* (PWB) and *Academic Buoyancy*. These findings are consistent with the literature, which shows that PWB and the ability to cope with daily academic challenges often go hand in hand, particularly through the role of positive emotions. Previous studies have reported that individuals with higher levels of well-being tend to report more positive emotions—such as enjoyment, hope, and optimism—which are also found to be associated with higher levels of buoyancy (Hirvonen et al., 2020; Zhou and Wang, 2024). Additionally, Academic Buoyancy has been associated with more adaptive emotion regulation, fewer negative emotional experiences, and more positive emotions in the learning process; these emotional patterns are associated with more adaptive learning behaviors, such as a tendency to make plans and a decrease in avoidance behaviors (Granziera et al., 2024; Hirvonen et al., 2020; Wang and Hui, 2024).

*The Broaden-and-Build Theory* framework (Fredrickson, 2001) provides a theoretical basis for understanding the coexistence of PWB, positive emotions, and more adaptive response patterns in an academic context. In this framework, PWB can be viewed as a *psychological resource* that often accompanies more conducive emotional and cognitive conditions when facing learning demands. These findings also align with the report by Saleem et al. (2022), which shows that positive psychological resources are associated with adaptive response patterns in the work context. However, the associations found in this study remain non-causal given the cross-sectional design.

### 4.2 H2—*Psychological Well-Being* and *Academic Gratitude*

The second finding shows that students with higher levels of *Psychological Well-Being* (PWB) tend to be better able to feel and express gratitude in an academic context. Theoretically, this relationship is consistent with the explanation that individuals with high PWB have more adaptive emotional regulation, making it easier for them to recognize and maintain positive emotions, including gratitude. This ability is also supported by a tendency to engage in *cognitive reappraisal*, which is interpreting experiences more positively, which in turn facilitates the emergence of gratitude for various experiences and academic support (Man and Jing, 2025).

In addition, high PWB provides a psychological foundation that makes individuals more sensitive to positive experiences and the kindness they receive. Students with healthy psychological conditions tend to form more supportive social relationships and engage in prosocial behavior, so they are more likely to experience and appreciate help or support in the academic environment. This pattern of positive interaction also reinforces the tendency for gratitude to emerge (Nawa and Yamagishi, 2024; Prathham Arora et al., 2023).

*The Broaden-and-Build Theory* framework (Fredrickson, 2001) further clarifies this mechanism. This theory argues that positive emotions broaden the mindset and increase cognitive flexibility, thereby creating internal conditions conducive to the development of appreciation and gratitude. Thus, high PWB can be understood as a foundation that enables students to be more open to positive experiences, more responsive to social support, and ultimately more capable of expressing *Academic Gratitude*. However, because the research design is cross-sectional, the direction of this relationship cannot be fully ascertained; a reciprocal relationship between PWB and gratitude remains possible and needs to be further tested through longitudinal or experimental studies.

### 4.3 H3—*Academic Gratitude* and *Academic Buoyancy*

The results of this study show that *Academic Gratitude* (AG) is positively related to *Academic Buoyancy*. Students who tend to appreciate the help, learning opportunities, and academic experiences they receive generally also report more adaptive responses when facing daily academic challenges. These findings are in line with previous reports showing that gratitude is associated with stronger perceptions of support and more stable positive emotion regulation, both of which are associated with better academic adaptation patterns (Demichelis et al., 2024; Theiyab Alazemi et al., 2023).

Previous studies have also shown that gratitude often goes hand in hand with resilience, a tendency toward *positive reframing*, and the use of adaptive coping strategies, enabling individuals to maintain an optimistic attitude in challenging situations (Pohan et al., 2025; Zainoodin et al., 2021). In the context of Indonesian higher education, Academic Gratitude can be reflected in appreciation for faculty support, supportive social relationships, and meaningful learning opportunities (Nisa, 2022; Pohan et al., 2025), all of which are relevant for understanding variations in students’ ability to respond to learning obstacles.

Overall, these patterns support the understanding that AG and Academic Buoyancy often appear together through psychological mechanisms such as positive emotion regulation, perceptions of social support, and a tendency to find positive meaning in academic experiences. However, the relationship found is associative and cannot be interpreted as causal.

### 4.4 H4—The mediating role of *Academic Gratitude*

The bootstrapping results show that *Academic Gratitude* (AG) partially mediates the relationship between *Psychological Well-Being* (PWB) and *Academic Buoyancy*. These findings indicate that gratitude can be one of the affective-cognitive pathways that help explain the relationship between positive psychological conditions and students’ ability to cope with academic challenges, in line with the *Broaden-and-Build Theory* framework (Fredrickson, 2001). However, all of the relationships found are correlational.

Previous literature indicates that individuals with higher PWB tend to report more experiences of gratitude in academic contexts (Buenconsejo et al., 2024; Kusumawati et al., 2023). AG itself is often associated with motivation, learning engagement, self-efficacy, resilience, and social support (Buenconsejo et al., 2024; Liu et al., 2024). These patterns are also reported to be related to Academic Buoyancy, including the ability to manage stress, maintain optimism, and use adaptive coping strategies (Cui et al., 2023; Li et al., 2022).

In summary, AG emerges as a mechanism that helps bridge the relationship between PWB and Academic Buoyancy, without implying a causal relationship.

### 4.5 Considerations regarding measurement quality

The evaluation of the measurement model indicates that all constructs exhibit adequate convergent validity and internal reliability, as indicated by AVE values ≥ 0.50 and CR values ≥ 0.85 (Hair et al., 2019).

However, the HTMT value for the *PWB–Academic Gratitude* construction pair is slightly above 1.0, indicating conceptual overlap between the two constructions. This may be due to the theoretical proximity between aspects of PWB (e.g., positive relationships, self-acceptance) and the tendency to feel grateful in an academic context. In addition, the use of self-report instruments may increase *the general method* var*iance*, thereby strengthening the correlation between constructs. In contrast, the other construct pairs (PWB–AB and AG–AB) showed HTMT values < 0.85, indicating adequate discriminant validity.

These findings confirm that empirical differences between PWB and AG should be interpreted with caution. In future research, multi-trait multi-method approaches, observational data, or third-party reports could be used to minimize single-method bias and clarify conceptual boundaries between constructs.

## 5 Theoretical and practical implications

The theoretical implications of these findings for educational psychology suggest that *Academic Gratitude* can be understood as an affective-cognitive mechanism that helps explain the relationship between *Psychological Well-Being* and *Academic Buoyancy*. These results expand the application *of Broaden-and-Build Theory* in higher education, positioning positive emotions—particularly gratitude—as a conceptual pathway to aspects of academic resilience. These findings also confirm that Academic Gratitude is a relevant stand-alone psychological construct, despite its empirical links to Psychological Well-Being.

Practically speaking, the results of this study open up opportunities to develop gratitude-based interventions as a supporting strategy to facilitate the adaptive aspects of learning activities. However, the effectiveness of these interventions still requires further testing through longitudinal or experimental designs to gain a stronger understanding of the relationships involved.

## 6 Limitations and directions for further research

This study has several limitations that need to be considered when interpreting the results. First, the cross-sectional design limits the ability to conclude the direction of the relationship between variables. Longitudinal or experimental studies are needed to test the reciprocal relationship between PWB, gratitude, and academic resilience.

Second, all data were collected via self-report instruments, which may introduce common-method *bias* and strengthen correlations among constructs, especially in the PWB-AG pair, which showed an HTMT value > 1.0. A multi-method or *multi-trait–multi-method* approach could be used in future studies to minimize this bias.

Third, the research sample came from a single university in West Java, so the findings should be generalized with caution. Future research could involve a more diverse sample from various institutions and regions.

Fourth, this study did not examine potential differences by individual characteristics, such as gender, study program, or semester level. Multi-group *SEM* analysis can be used to examine the role of moderator factors in the relationships among variables.

Fifth, although all constructs show adequate convergent validity, the empirical overlap between PWB and AG indicates the need for further testing to clarify the conceptual boundaries of both variables in the Indonesian cultural context. Non-self-report assessment approaches or the integration of objective data (e.g., academic records) may provide a more comprehensive understanding.

Sixth, the discussion of cultural context is interpretive because no cultural variables were directly measured in this study.

## 7 Conclusion

This study shows that *Psychological Well-Being*, *Academic Gratitude*, and *Academic Buoyancy* are interrelated among the students who participated. *Academic Gratitude* acts as a partial mediator in the relationship between *Psychological Well-Being* and *Academic Buoyancy*, *as indicated by statistical analyses*, without implying a causal relationship. Although these findings do not imply a causal relationship, this study’s results provide a deeper understanding of the emotional mechanisms that support student academic resilience and open up opportunities for the development of positive emotion-based interventions in higher education.

## Data Availability

The raw data supporting the conclusions of this article will be made available by the authors, without undue reservation.
